# Predictors of time to first symptomatic recovery of major depressive disordered patients: a case study at Jimma University Medical Center

**DOI:** 10.1186/s12888-022-04443-8

**Published:** 2023-01-13

**Authors:** Ketema Zerihun Asefa, Tadele Degefa Bedada, Jaleta Abdisa Fufa, Firomsa Shewa Gari, Gurmessa Nugussu Gelcho, Geremew Muleta Akessa

**Affiliations:** 1 Department of Statistics, College of Natural and Computational Science, Madda Walabu University, Bale-Robe, Oromia Ethiopia; 2 Department of Statistics, College of Natural Science, Jimma University, Jimma, Oromia Ethiopia; 3 Department of Statistics, College of Natural and Computational Science, Assosa University, Assosa, Benishangul Gumuz Ethiopia

**Keywords:** Major depressive disorder, First symptomatic recovery, Gamma, Inverse gaussian, Frailty, Weibull, Log-logistic, Log-normal, Akaike information criteria

## Abstract

**Background:**

Major Depressive Disorder is one of the most common mental disorders, and it is the main cause of disability worldwide with a prevalence ranging from 7 to 21%.

**Objective:**

The goal of this study was to predict the time it took for patients with severe depressive disorders at Jimma University Medical Center to experience their initial symptomatic recovery.

**Study design:**

The researchers utilized a prospective study design.

**Methods:**

Patients with major depressive disorder were followed up on at Jimma University Medical Center from September 2018 to August 2020 for this study. The Gamma and Inverse Gaussian frailty distributions were employed with Weibull, Log-logistic, and Log-normal as baseline hazard functions. Akaike Information Criteria were used to choose the best model for describing the data.

**Results:**

This study comprised 366 patients, with 54.1% of them experiencing their first symptomatic recovery from a severe depressive disorder. The median time from the onset of symptoms to symptomatic recovery was 7 months. In the study area, there was a clustering effect in terms of time to first symptomatic recovery from major depressive disorder. According to the Log-normal Inverse-Gaussian frailty model, marital status, chewing khat, educational status, work status, substance addiction, and other co-variables were significant predictors of major depressive disorder (p-value < 0.05).

**Conclusion:**

The best model for describing the time to the first symptomatic recovery of major depressive disorder is the log-normal Inverse-Gaussian frailty model. Being educated and working considerably were the variables that reduces the time to first symptomatic recovery from major depressive disorder; whereas being divorced, chewing khat, substance abused and other co-factors were the variables that significantly extends the time to first symptomatic recovery.

## Introduction

Major depressive disorder (MDD) is one of the most common mental diseases in the world and a leading cause of disability [1]. The prevalence of this disorder varies between 7 and 21% [2]. Depression is the second greatest cause of disability worldwide, with a little more than 4% of the global population suffering from it. Afghanistan has the highest rate of depression, with more than one in every five people suffering from it, while Japan has the lowest rate (2.5 percent) [3]. According to global disease burden estimates, depressive episodes affect 5.8% of men and 9.5% of women worldwide [4]. If current trends continue, depression will account for 5.7% of the overall illness burden by 2030, ranking it second only to heart disease [4].

There are various depression prevalence rates throughout Africa. Depression affects more than 5% of the population in Sub-Saharan African countries [3]. For example, the prevalence of depression in South Africa was 9.7% throughout a lifetime and 4.9% in the 12 months before the interview [5], while it was 5.2% in Nigeria [5]. According to a 2012 report from the Ethiopian Federal Ministry of Health, the prevalence of depression in Ethiopia was 5%, while a WHO survey conducted in partnership with Jimma University found that the prevalence of MDD in Ethiopia was 9.1% [6, 7]. According to a national survey conducted in 2014, the combined prevalence of MDD in Ethiopia was 11% [8].

Major Depressive Disorder is a common, often chronic, and recurrent mental disorder marked by persistent unhappiness and ill health. A mood disorder induced by a mental ailment is known as major depression disorder, often known as unipolar or clinical depression disorder. It has a negative impact on emotional, intellectual, vocational, and family functioning [1]. MDD is characterized by depressed mood, lack of interest and enjoyment, decreased energy, melancholy, tenseness, irritability, feelings of grief or low self-worth, and disturbed sleep [2, 9]. It is diagnosed when a person has a persistently low or depressed mood, decreased interest in enjoyable activities, feelings of guilt or irrelevance, lack of energy, poor attention, enthusiasm changes, psychomotor delay or anxiety, sleep instabilities, or hopeless thoughts [10, 11].

One of the most treatable mental illnesses is major depressive disorder. Between 80 and 90% of depressed patients eventually benefit from therapy. Almost all patients experience some symptom relief. MDD is initially treated with either medication or psychotherapy. Psychotherapy and medicine together have been demonstrated to be more successful than any of these treatments separately [12]. For severe major depression, electroconvulsive therapy has been demonstrated to be more effective than all other treatments combined [13]. Even severe depression responds well to treatment. Antidepressant medications, psychotherapy, or a combination of the two are the most common treatments for depression. One or both of these treatments may be beneficial in mild or moderate depression, although medication is often suggested as a first step in the treatment of severe or incapacitating depression. In a combined treatment, drugs can swiftly alleviate physical symptoms, while psychotherapy allows patients to learn more effective problem-solving techniques. Antidepressants are medications that are used to treat depression such as selective serotonin reuptake inhibitors (SSRIs) and tricyclics and monoamine oxidase inhibitors (MAOIs) [12].

Recovery in patients with MDD is associated with improvement on multiple outcome domains. Symptom severity and acceptance showed the strongest association with perceived well-being [14]. For many people with mental illness, the concept of recovery is about staying in control of their life rather than the elusive state of return to premorbid level of functioning. Many factors are associated with the road to recovery and include good relationships, financial security and satisfying work [15–17]. The environment, which provides for personal growth, developing resilience to stress and adversity and allows people to develop cultural and spiritual perspectives, is also crucial. Being believed in, listened to and understood by families, friends and health and social service personnel are very helpful to people on the road to recovery. Getting explanations for problems or experiences and developing skills and receive support to achieve their goals are crucial to success. Support during periods of crisis is also critical.

Obstacles to adherence include poor tolerability, social stigma, inadequate patient education, lack of patient motivation, concerns about medication cost, weight gain, sexual dysfunction, delayed onset of efficacy, failure of patients to perceive benefits of treatment, and premature discontinuation of treatment after symptoms have improved [18–21]. Similarly, factors found to hinder recovery from mental health difficulties including social exclusion, discrimination, inaccessibility to work, and economic hardship [22, 23] might also hinder patients’ recovery from depression. This is in line with other studies in which comorbid medical conditions as well as psychiatric illnesses such as anxiety disorder, dysthymia, personality disorder, and substance abuse exerted a negative effect on the course of depression [24].

Major depressive disorder patients who achieved recovery (52.1%) were significantly less likely to have impaired levels of functioning, concurrent medical or psychiatric conditions, low levels of education, or non-adherence to therapy at follow-up. The level of functioning during the index episode seems to be a better predictor of recovery than symptom severity. Therefore, the level of functioning should be considered while determining recovery from depression [25]. Increased likelihood of recovery is associated with less severe depressive symptoms, lower anxiety scores, and lower levels of personality dysfunction [18, 19], whereas factors such as lower economic status, measured by education, income, or occupation, concurrent psychiatric and medical conditions, longer duration of index episode, and older age are associated with a decreased likelihood or delayed achievement of clinical remission [17]. Thus, the present study aimed to study the factors affecting the duration of first symptomatic recovery from MDD in the study area.

Data that measures the time to a certain event of interest is referred to as survival data [26]. The event of interest in this study was the first symptomatic recovery from MDD after therapy. The Cox proportional hazards model does not account for survival data heterogeneity [27]. As a result, the shared frailty model uses unbiased parameter estimates to address any heterogeneity and random effects [28–30]. Jimma town, where primary health care is provided and mental health services are decentralized, has a high rate of mental distress [31]. As a result, we used a shared frailty model to analyze the characteristics related with time to first symptomatic recovery from MDD while accounting for data heterogeneity.

The present study plays very important roles in psychiatry department of the study area; because it is one way of overcoming the mental health problems in the community by identifying the significant determinants of recovery duration from MDD. Although the detrimental impact of major depressive disorder (MDD) at the individual level has been described, its local epidemiology remains unclear given limitations in the data. Here, we present the modeled epidemiological profile of MDD dealing with heterogeneity in the districts, enforcing internal consistency between epidemiological parameters and making estimates for world regions with no empirical data. These estimates were used to quantify the burden of MDD for the study area and for the Global Burden of Disease Study as well. This has more advantage for health professionals and psychiatrists in order to give the appropriate treatments for the MDD patients using identified risk factors as a baseline. It also helps physicians and researchers as a landmark for further studies related to MDD and other mental disorders.

## Methods

### Source of data and study design

The data for this study came from the Jimma University Medical Center, which is located in the Jimma Zone of Oromia Regional State in Ethiopia’s south west. Jimma Zone is approximately 325 km from Ethiopia’s capital city, Addis Ababa.

The patient’s registry dates to the event time or censoring time in this data, which is secondary data recorded at the hospital. As a result, after identifying patients who were admitted and followed up from September 1, 2018 to August 31, 2020, data was retrieved from the patient’s card, which contains epidemiological, laboratory, and clinical information of MDD patient’s card and information sheet. The first symptomatic recovery, which was otherwise censored, was the event for this investigation. The information on the suppressed or abridged subjects, however, is incomplete. Patients with MDD who did not have symptomatic recovery over the research period, lost, or withdrew before symptomatic recovery were censored. Patients who were admitted for follow-up of all major depressive disorders for at least three visits at Jimma University Medical Center from September 2018 to August 2020 were included in this study, which used a prospective cohort study design. A total of 366 patients with depression disorders were enrolled in this investigation.

### Variables in the study

The survival time (time to first symptomatic recovery) evaluated in months from the start of treatment to the date of the patient’s recovery or censored was the dependent variable in this study. The patients' status was 1 if they recovered and 0 if they were censored during the study period. About the recovery, the psychiatrist made decision based on the psychiatric examination. The standards criterion is by using the DSM-5 diagnostic criteria when the patient is fully free from those symptoms for at least six months. There are different instrument, especially regarding to screening the patient sign and symptom to know whether suffering from specific mental illness or psychological distress, but there are only to confirming or what you call diagnostic instrument. Those are: 1) DSM-5 which stands for Diagnostic Statistical Manual version five and 2) ICD-11 which stand for International Classification of Disease version 11 for which in Ethiopia we use DSM-5.

Major depressive episode was diagnosed when at least 2 weeks of persistent depressed mood, anhedonia, or hopelessness occurred (reported by self or observed by others), plus additional symptoms from criterion A, for a total of 5 of the 9 DSM-5 major depression criteria [32] and the clinical significance criterion. Lifetime DSM-5 MDD was defined as at least one lifetime major depressive episode without full DSM-5 manic, mixed, or hypomanic episodes, [32, 33] excluding substance induced and medical-induced disorders. Those with at least one episode in the prior 12 months were classified as having 12-month MDD. Clinical validity was assessed through concordance with blinded clinician reappraisals using the Psychiatric Research Interview for Substance and Mental Disorders, DSM-5 version (PRISM-5) [34, 35]. Concordance for binary MDD diagnoses was fair [36] (*κ *= 0.35–0.46) and higher with corresponding DSM-5 MDD dimensional scales (intraclass correlation, 0.60–0.64) [34].

Gender, age, marital status, first onset age, educational status, other cofactors, family history of mental illness, substance abuse, religion, ethnicity, chewing khat, and employment status were all considered factors of recovery duration (independent variables) in the study.

### Inclusion and exclusion criteria

All patients (12–65 years old) with major depressive disorder were included in the study**,** whereas children under the age of 12, pregnant or lactating women (less than 6 months), and patients with irrelevant information during the study period were excluded. MDD is less common in pre-school children (1–2%) than in adults (20%) [37], hence children under the age of 12 were excluded.

### Statistical methods

Data that measures the time to a certain event of interest is referred to as survival data [26]. Estimates of the survival function and hazard function are useful for summarizing survival data. Because no explicit assumptions regarding the underlying distribution of survival times are required, this method is non-parametric or distribution frees [38]. Otherwise, survivor function estimators, such as the Kaplan–Meier (KM) survival function estimator and the log-rank test for comparing two or more groups of categorical variables, were utilized in this work.

Suppose we have a sample of independent observations, their survival times denoted by ${t}_{1}, {t}_{2}, {t}_{3}, ..., {t}_{n}$ and indicators of censoring denoting by ${\delta }_{1}, {\delta }_{2}, {\delta }_{3}, ..., {\delta }_{n}$ where

$${\delta }_{i}=\left\{\begin{array}{l}1, if\,the\,first\,symptomatic\,occur\\ 0, otherwise\end{array}\right.$$

Thus, the survival data are denoted by ${t}_{i}, {\delta }_{i}; i=1, 2, 3, .., n$. The first step to obtain the KM estimator of the survival function is to order the survival times as ${t}_{1}, {t}_{2}, {t}_{3}, ..., {t}_{n}$. Assume that $m\le n$ events occurred at distinct m times among the n observations. The probability that an event will not occur by time t:S(t) = P(T > t) is the main quantity of interest. The survival function is estimated by Kaplan and Meier.

$${\widehat{S}}_{KM}\left(t\right)=\prod_{{t}_{i}\le t}{\left(\frac{{n}_{i}-{d}_{i}}{{n}_{i}}\right)}^{{\delta }_{i}}=\prod_{{t}_{i}\le t}{\left(1-\frac{{d}_{i}}{{n}_{i}}\right)}^{{\delta }_{i}},$$

where ${d}_{i}$ is number of patients experienced event at ${t}_{i}$ and ${n}_{i}$ is number of patients at risk before ${t}_{i}$ [38, 39].The log-rank test which is used for comparison of the survival curves of two or more categorical covariates also applied [40].

A random effects model with shared frailties is one in which the frailties are common (or shared) among groups of individuals or spells and are randomly distributed among groups. The shared frailty model is a conditional model in which all participants in a cluster share frailty [41, 42]. The multivariate frailty model is a variation of the univariate frailty model that permits people in the same cluster to have the same frailty value.

The researchers assumed that there is a clustering (frailty) effect on modeling time-to-first symptomatic recovery from MDD which might be due to the heterogeneity in district from which the patients came-from i.e. patients’ coming from the same district share similar risk factors related to MDD. Clusters with minimum median time have smaller frailties, so that these clusters are predicted to have a high hazard and more probable to first symptomatic recovery [43]. These nuisance terms modify the hazard function, so that the hazard function should be evaluated conditionally on this effect. Moreover, districts frail more are more likely to symptomatic recovery than the less frail districts (since the event is positive).

Conditional on the random term, called the frailty denoted by ${u}_{i}$, the survival times in cluster $i (1\le i\le n)$ are assumed to be independent, the proportional hazard frailty model assumes.

$${h}_{ij} \left(\frac{t}{{X}_{ij}}, ui\right)= exp\left({\beta }^{\mathrm{^{\prime}}}{X}_{ij}+{u}_{i}\right){h}_{0}\left(t\right),$$

where ${u}_{i}$ the random term of all the subjects in cluster.

The choice of frailty distribution is critical for obtaining an accurate description of the data’s dependent structure. Gamma and Inverse Gaussian frailty distributions were used in this investigation. In both cases, the degree of independence is represented by a single heterogeneity parameter (denoted by θ).

The functional form of the one parameter gamma distribution is given by:

$${f}_{z}\left({Z}_{i}\right)= \frac{{{Z}_{i}}^{(\frac{1}{\theta })-1}\mathrm{exp}(-{Z}_{i}/\theta )}{\Gamma (\frac{1}{\theta }){ \theta }^{{}^{1}\!\left/ \!{}_{\theta }\right.}},\theta >0$$

The inverse Gaussian (inverse normal) distribution was introduced as a frailty distribution alternative to the gamma distribution by [44]. The probability density function of an inverse Gaussian shared distributed random variable with parameter $\theta > 0$ is given by:

$${f}_{z}\left({Z}_{i}\right)=( {\frac{1}{2\pi \theta })}^\frac{1}{2}{Zi}^{-\frac{3}{2}}{\mathrm{exp}\left(\frac{-\left(zi-1\right)}{2\theta zi}\right)}^{2},\theta >0,z>0$$

The baseline hazard functions for the parametric shared frailty models were the Exponential, Weibull, and Log normal distributions.

Furthermore, the Akaike Information Criterion (AIC) was utilized to choose the optimal model for describing the data. Quantile–Quantile plots were used to examine the goodness of the fitted model, whereas Cox-Snell residuals were used to evaluate the baseline parameters. The data was analyzed using R-3.6.3 program.

## Results

### Descriptive summary of characteristics of patients

From September 2018 to August 2020, 366 patients with major depressive disorder at Jimma University Medical Center were enrolled in this study (Table 1). The event occurred in 54.1 percent of the 366 MDD patients (first symptomatic recovery from MDD). Patients' median symptomatic recovery duration was assessed to be 7 months. The majority of patients (51.1%) were men, with 41.2 percent of males experiencing symptomatic recovery. Females, on the other hand, experienced symptomatic recovery in 67.6% of cases. Male and female symptomatic recovery times were 11 and 9 months, respectively.

**Table 1:** Result from descriptive summary of characteristics of MDD patients

Variable	Categories	Censored *n* (%)	Events *n* (%)	Total *n* (%)	Median Time in Months (95% CI)
Gender	Male	110(58.8%)	77(41.2%)	187(51.1%)	11 (9,18)
	Female	58(32.4%)	121(67.6%)	179(48.9%)	9 (5, 13)
Age	13–19	15(40.5%)	22(59.5%)	37(10.1%)	6 (5, 20)
	20–25	35(35.7%)	63(64.3%)	98(26.8%)	7 (5, 8)
	26–49	52(36.1%)	92(63.9%)	144(39.3%)	6 (5, 9)
	≥ 50	66(75.9%)	21(24.1%)	87(23.8%)	13 (12, 14)
Marital status	Single	38(34.5%)	72(65.5%)	110(30.1%)	6 (5, 9)
	Married	38(27.9%)	98(72.1%)	136(37.2%)	5 (4, 7)
	Widowed	50(80.6%)	12(19.4%)	62(16.9%)	21 (20, 22)
	Divorced	42(72.4%)	16(27.6%)	58(15.8%)	19 (13, 22)
First onset age	Childhood	8(40%)	12(60%)	20(5.5%)	11 (5, 12)
	Adolescent	78(39.4%)	120(60.6%)	198(54.1%)	7 (6, 9)
	Adult	82(55.4%)	66(44.6%)	148(40.4%)	9 (7, 18)
Family history	No	67(35.3%)	123(64.7%)	190(51.9%)	6 (5, 7)
	Yes	101(57.4%)	75(42.6%)	176(48.1%)	12 (9, 18)
Chewing khat	No	53(23%)	177(77%)	230(62.9%)	5 (4, 6)
	Yes	115(84.6%)	21(15.4%)	136(37.1%)	13 (11, 20)
Educational status	Uneducated	152(70.7%)	63(29.3%)	215(58.7%)	13 (12, 15)
	Educated	16(10.6%)	135(89.4%)	151(41.3%)	9 (3, 15)
Employment	No	105(60.3%)	69(39.7%)	174(47.5%)	9 (8 19)
	Yes	63(32.8%)	129(67.2%)	192(52.5%)	6 (4, 7)
Religion	Orthodox	47(49.5%)	48(50.5%)	95(25.9%)	9 (6, 18)
	Muslims	90(43.7%)	116(56.3%)	206(56.3%)	10 (6, 11)
	Protestant	24(52.2%)	22(47.8%)	46(12.6%)	7 (6, 8)
	Others	7(36.8%)	12(63.2%)	19(5.2%)	6 (4, 7)
Ethnicity	Oromo	112(46.3%)	130(53.7%)	242(66.1%)	8 (6, 11)
	Amhara	42(49.4%)	43(50.6%)	85(23.2%)	7 (6, 18)
	Others	14(35.9%)	25(64.1%)	39(10.7%)	6 (5, 13)
Substance	No	61(32.1%)	129(67.9%)	190(51.9%)	5 (4, 6)
	Yes	107(60.8%)	69(39.2%)	176(48.1%)	12 (10, 18)
Other Cofactors	No	40(19.8%)	162(80.2%)	202(55.2%)	5 (4, 6)
	Yes	128(78%)	36(22%)	164(44.8%)	10 (9, 22)
Event of Relapse	No	64(33.7%)	126(66.3%)	190(51.9%)	6 (5, 7)
	Yes	104(59.1%)	72(40.9%)	176(48.1%)	13 (5, 17)

Individuals who have abused substances have a longer survival time to first symptomatic recovery than patients who have not consumed substances (Table 2). Individuals who are educated had a shorter time to initial symptomatic recovery than patients who are illiterate. This indicates that educated people recovered from their symptoms faster than illiterate patients. Individuals who were employed had a shorter time to initial symptomatic recovery than patients who were unemployed. Patients who chew khat have a longer survival time to first symptomatic recovery than those who do not chew. Patients who had other cofactors had a longer survival time to symptomatic recovery than patients who did not have other cofactors.

**Table 2:** Result from Log-rank test

Variables	Category	Chi-square	df	Sig
Marital Status	Single			
	Married			
	Widowed			
	Divorced	39.1	3	< 0.001
Khat Chewing	No			
	Yes	85.7	1	< 0.001
Educational Status	Uneducated			
	Educated	77.1	1	< 0.001
Employment Status	No			
	Yes	21.1	1	< 0.001
Substance Abuse	No			
	Yes	25.9	1	< 0.001
Other Cofactors	No			
	Yes	75.6	1	< 0.001

### Results from univariable analyses and model comparison

The significance level for the univariable analysis was set at 25%. In the multivariable analysis, all significant factors from the univariable analysis were included. The Weibull, Log-logistic, and Log-normal hazard functions were used as the baseline hazard functions, with Gamma and Inverse Gaussian frailty distributions. When compared to other models, the Lognormal-Inverse-Gaussian model had the lowest AIC value (Table 3). As a result, the lognormal-inverse Gaussian model was the best fit for the data in this investigation.

**Table 3:** Result from parametric frailty models comparison

Baseline hazard function	Frailty distribution	AIC
Weibull	Gamma	1197.7
	Inverse-Gaussian	1192.3
Log-logistic	Gamma	1183.9
	Inverse-Gaussian	1174.9
Log-normal	Gamma	1181.9
	Inverse-Gaussian	1172.5

### Results from multivariable analyses

At a 5% level of significance, the Lognormal-Inverse-Gaussian frailty model revealed that marital status, khat chewing, educational level, job, substance misuse, and other cofactors were important determinants of MDD patients (Table 4).

**Table 4:** Result from Lognormal-inverse Gaussian multivariable analysis

Covariates	Category	Coef	S.E	ɸ	95% CI	p-value
Gender	Male	Ref		1		
	Female	-0.149	0.129	0.862	[0.609, 1.114]	0.25
Age of patients	13–19	Ref		1		
	20–25	-0.057	0.215	0.944	[0.523, 1.366]	0.79
	26–49	-0.031	0.211	0.969	[0.556, 1.383]	0.88
	≥ 50	0.38	0.255	1.471	[0.962, 1.962]	0.13
Marital status	Single	Ref		1		
	Married	-0.212	0.143	0.808	[0.529, 1.089]	0.14
	Widowed	0.3215	0.240	1.379	[0.909, 1.849]	0.18
	Divorced	0.6195	0.230	1.858	[1.407, 2.309]	0.0071
Family History	No	Ref		1		
	Yes	0.1419	0.132	1.1523	[0.894, 1.411]	0.28
Chewing Khat	No	Ref		1		
	Yes	0.9028	0.174	2.466	[2.125, 2.807]	≤ 0.001
Educational Level	Uneducated	Ref		1		
	Educated	-0.517	0.138	0.596	[0.323, 0.867]	≤ 0.001
Employment	No	Ref		1		
	Yes	-0.4179	0.129	0.658	[0.406, 0.911]	0.0012
Substance Abuse	No	Ref		1		
	Yes	0.3966	0.134	1.487	[1.224, 1.749]	0.003
Other Cofactors	No	Ref		1		
	Yes	0.4905	0.151	1.633	[1.337, 1.929]	0.0011
Event of Relapse	No	Ref		1		
	Yes	0.2058	0.132	1.228	[0.969, 1.487]	0.12
	θ = 0.21	τ = 0.081		AIC = 1172.54		

In this study, patients’ marital status had a significant impact on the first symptomatic recovery of MDD patients; the acceleration factor of divorced patients was 1.858 times higher than single patients (ɸ = 1.858, 95 percent CI: 1.407, 2.309), implying that divorced patients had a 1.858-fold longer symptomatic recovery time from MDD than single patients.

Similarly, khat chewing was the most important factor in MDD patients' first symptomatic recovery; the acceleration factor of patients who chewed khat was 2.466 times higher than that of patients who did not chew khat (ɸ = 2.466, 95 percent CI: = 2.125, 2.807), indicating that patients who chewed khat had a symptomatic recovery time from MDD that was 2.466 times longer than those who did not chew khat.

Regarding education status, the acceleration factor of educated patients was 0.596 times smaller than that of patients with no education (ɸ = 0.596, 95 percent CI: 0.323, 0.867); this means that the symptomatic recovery time of educated patients was 40.4 percent shorter than that of patients with no education.

Employment status was another covariate that had a significant impact on patients' symptomatic recovery time; the acceleration factor of employed patients was 0.658 times less than that of unemployed patients (ɸ = 0.6580, 95 percent CI: 0.406, 0.911), indicating that employed patients' symptomatic recovery time was reduced by 34.2 percent when compared to unemployed patients.

According to the findings of this study, substance usage had an effect on the first symptomatic recovery MDD patients. The acceleration factor of substance-abusing MDD patients was 1.487 times higher than that of non-abusing MDD patients (ɸ = 1.487, 95 percent CI: 1.224, 1.749), implying that substance-abusing patients had a 48.7% shorter survival time than non-abusing patients. When other cofactors were considered, patients with other cofactors had a 1.663 longer first symptomatic recovery of MDD than those without (ɸ = 1.633, 95 percent CI: = 1.337, 1.929).

In the lognormal-inverse Gaussian frailty model, the form parameter is equal to 3.56, indicating that the hazard function is unimodal (i.e., it increases up to some time and then decreases). The district’s heterogeneity was calculated to be 0.21, and the district’s reliance was estimated to be around 8.1 percent.

**Fig. 1:**
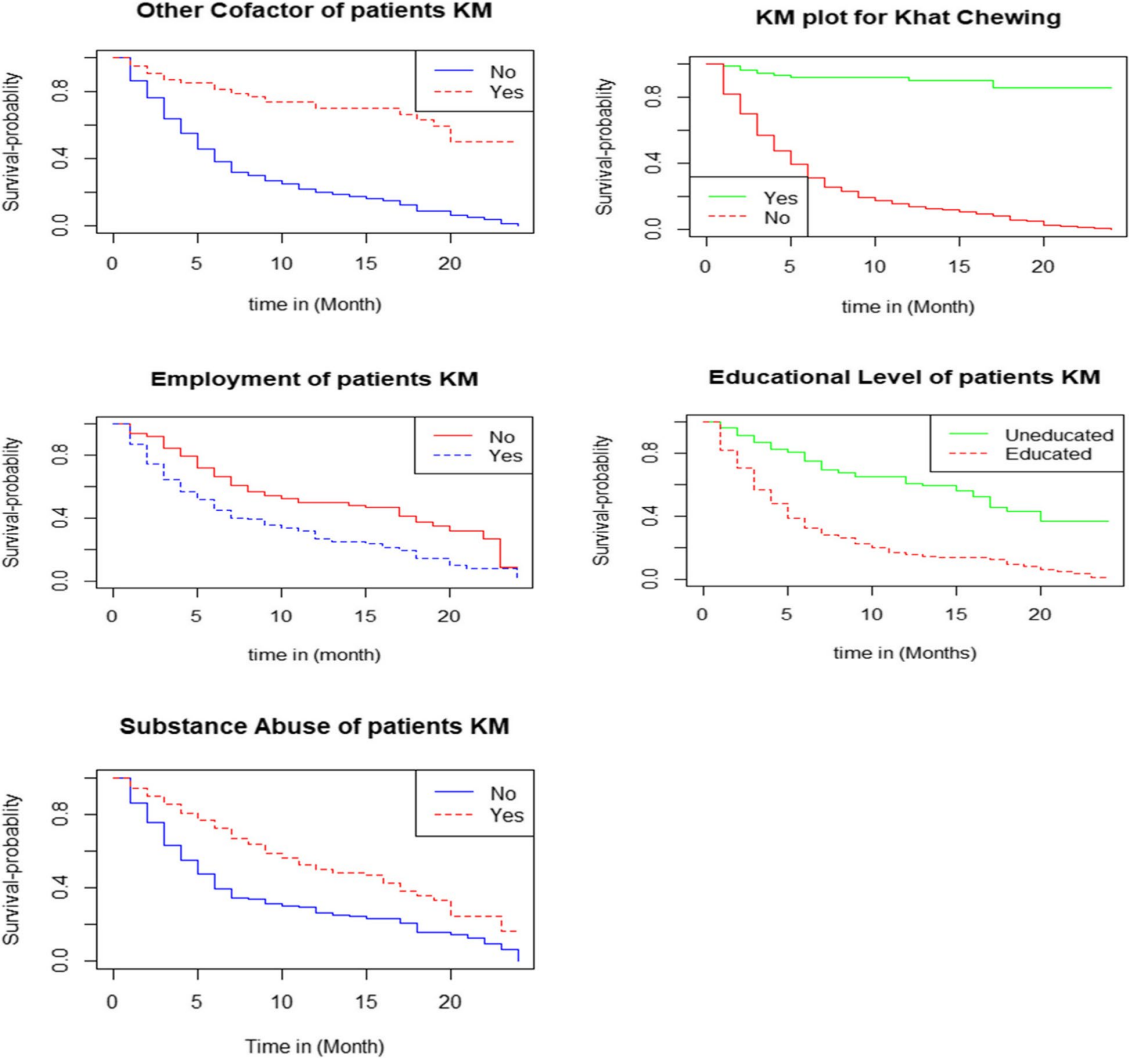
The survival functions of the categories of independent variables

**Fig. 2:**
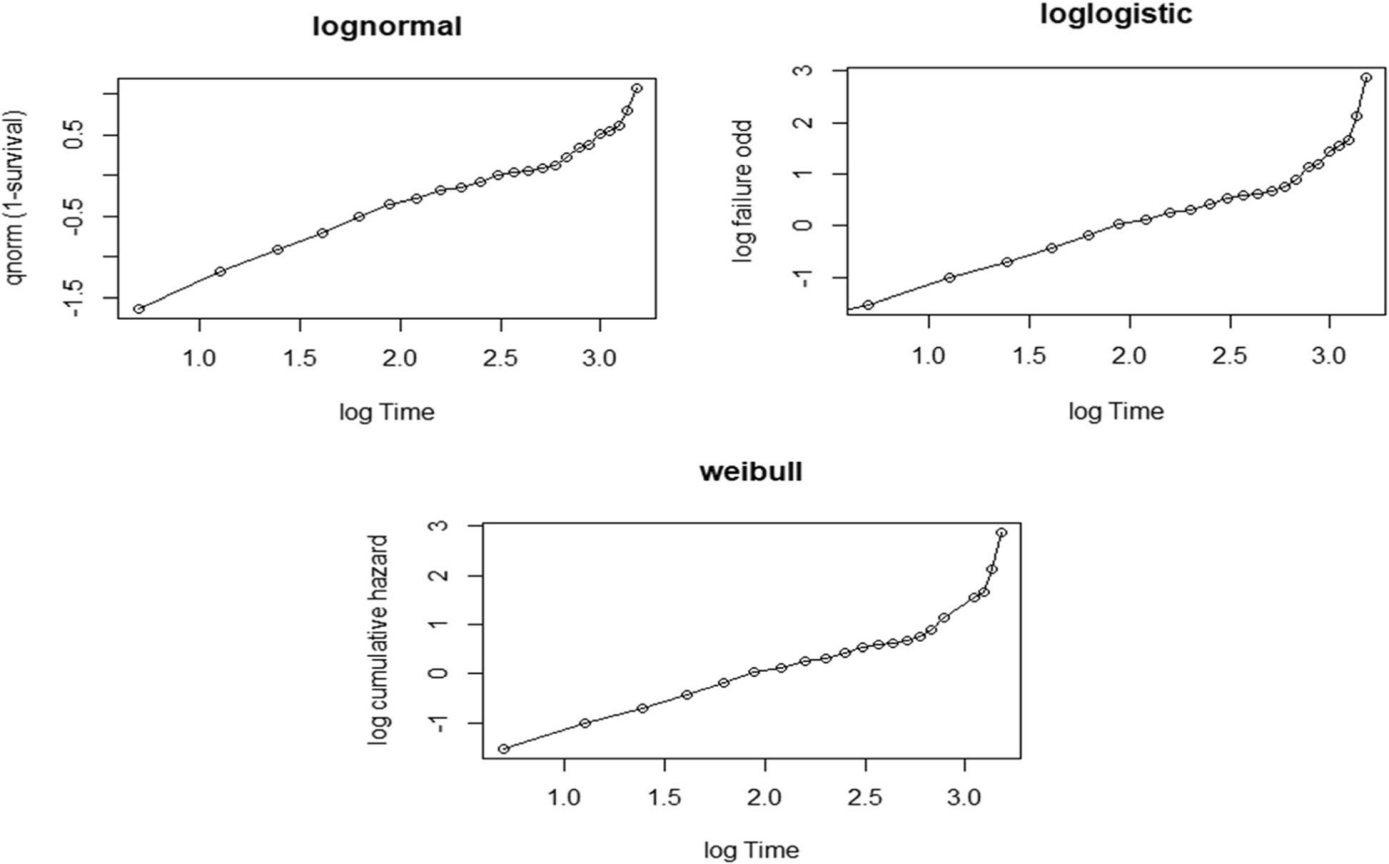
Graphical evaluation of the Weibull, Log-logistic and Log-normal assumptions

The Weibull has been displayed using the logarithm of cumulative hazard function with the logarithm of time-to-recovery from MDD to assess the adequacy of our baseline hazard (Fig. 1). Similarly, the logarithm of the failure chances has been plotted against the logarithm of time-to-recovery from MDD, and the log-normal has been plotted against the logarithm of time-to-recovery from MDD (Fig. 2). The log-normal plot was more linear than the other plots, indicating that the log-normal model is superior to the others.

**Fig. 3:**
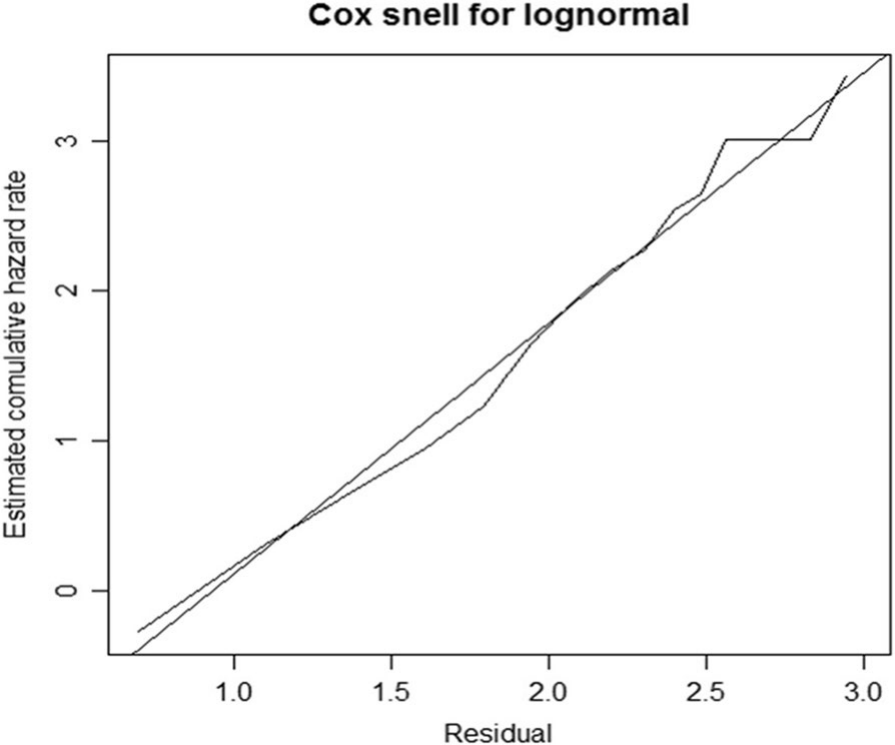
Cox-Snell residuals obtained by fitting log-normal to the MDD patients’ dataset

The cumulative hazard function of the Cox-Snell residuals with Weibull, Log-logistic, and Log-normal models was plotted, revealing that the Log-normal model was closest to the line through the origin as compared to the other models, implying that the Log-normal model accurately describes the MDD dataset (Fig. 3).

**Fig. 4:**
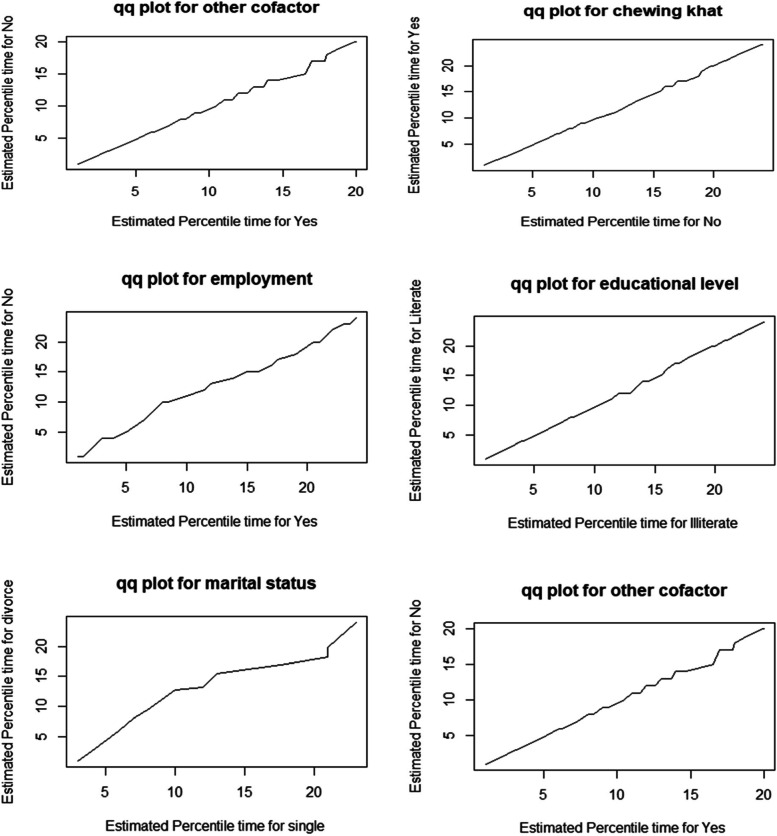
Q-Q plots to check the adequacy of accelerated failure time model

A Quantile–Quantile plot is used to see if the accelerated failure time provides a good fit to the data for two different demographic groups. We compared the significantly varied educational levels, employment status, marital status, chewing khat, other cofactors, and substance misuse, which indicate linear for all significant covariates, to assess the adequacy of the accelerated failure time model graphically (Fig. 4).

## Discussion

In this study, 366 patients with major depression disorder were enrolled; 54.1% of them had their first symptomatic recovery confirmed, while 45.9% were censored. This finding is consistent with a recent study by Novic et al., which found 52.1% symptomatic recovery and 47.9% non-recovery among the patients studied [45].

The authors checked for heterogeneity within clusters (district), which was significant and estimated to be 0.21, whereas cluster dependence is about 0.081 (8.1%), indicating that there is a larger degree of heterogeneity across district and substantial relationship within district.

The outcomes of this study demonstrated that education and employment greatly speed-up the time to first symptomatic recovery from MDD, but divorce, chewing khat, substance abuse, and other cofactors significantly slow down the time to first symptomatic recovery from MDD.

The Lognormal-Inverse-Gaussian shared frailty model with the lowest AIC value is the best model for fitting the data. According to the findings, there was a clustering (frailty) influence on the time to first symptomatic recovery from MDD. This could be owing to the district’s heterogeneity (i.e., patients coming from the same district share similar risk factors related to MDD). The findings of this study revealed that the patient’s educational level had a substantial impact on the time it took for them to experience their initial symptomatic recovery from MDD. Patients with education had a 0.596 times higher chance of symptomatic improvement from MDD than those with no education. This result is consistent with research conducted in South Africa and Turkey [45–47]. This could be because people without an education are valued less for their self-esteem and live more stressful lives than those who are educated. Furthermore, when compared to the uneducated, educated people had a greater understanding of the elements that contribute to depression.

The marital status of the patients had a positive impact on the time to first symptomatic recovery from MDD in the study area. When compared to patients with a single marital status, divorced patients had a longer (ɸ = 1.858) time to first symptomatic recovery from MDD. The high prevalence of major depression in separated or divorced individuals is due to both an increased risk of marital disruption in those with major depression, and also to the higher risk of this disorder in those with divorced or separated marital status [48]. The current study is comparable to the one published in [49–52].

In addition, chewing khat is a risk factor for MDD, according to the findings of this study. Damena et al. conducted research at Jimma University, which found that depression was substantially connected with chewing khat, and that the likelihood of experiencing depression episodes among khat chewers is tenfold more than that of non-chewers [53].

Also, the findings of this study revealed that patients’ work situation had a significant impact on the time it took for them to experience their initial symptomatic recovery from MDD. When compared to unemployed patients, employed individuals had less time to recover from MDD (ɸ = 0.658). This conclusion is in line with research conducted in the United States and Ethiopia [46, 54]. Substance misuse has also been established as a predictor of first symptomatic recovery from MDD. Individuals who used substances were (ɸ = 1.487) less likely to recover from MDD than patients who did not use substances. A study conducted in the Mekelle general jail center [55] supports this finding. Moreover, this conclusion is consistent with prior findings suggesting an association between higher levels of substance use and higher levels of MDD [56].

Furthermore, the findings of the study revealed that other patient cofactors had a substantial impact on the time to symptomatic recovery from MDD. Patients who had other cofactors had a recovery period that was 1.633 times longer than those who did not. The findings are consistent with those of Egede’s study, which found that the prevalence and risk of depression are significant among individuals with chronic medical disorders [57, 58]. Similarly, a study conducted in Ethiopia [11] corroborated the findings.

## Limitations

As a result, there are numerous predictive indicators for MDD recovery; nevertheless, the study was confined to only thirteen covariates. Because the patient’s card contains features that are unrelated to MDD recovery, as well as certain significant factors such as economic position, social relationships, loneliness, and health-related issues, the patient’s card is incomplete. Insufficient information about the precise details of how recovery was assessed and to some extent the heterogeneity of time between assessments across selected patients was also the challenges that the authors faced. Moreover, as a result of the absence of earlier research studies on this topic and the abundance of literature, these are the expected risk variables.

## Conclusion

The Lognormal-Inverse-Gaussian frailty model best describes the period to initial symptomatic recovery of patients with major depressive disorder. The results of the Lognormal-Inverse-Gaussian shared frailty model revealed that marital status, khat chewing, employment status, educational level, substance addiction, and other cofactors were all significant predictors of time to first symptomatic recovery in patients with severe depressive illness. The median period from the onset of symptomatic recovery in patients with major depressive disorder was seven months. Because of the variability between the district, there is a fragility (clustering) effect on the time to first symptomatic recovery from serious depressive illnesses. Patients who have taken a long time to recover should be treated appropriately by health experts (physicians) based on the risk factors identified.

## Data Availability

The data that support the findings of this investigation are accessible from the corresponding author; however, they are subject to restrictions because they were used under permission for the current study and are therefore not publicly available.

## References

[1] Depression WH. Other common mental disorders: global health estimates. Geneva: World Health Organization. 2017. p. 24.

[2] Kessler RC, Bromet EJ (2013). The epidemiology of depression across cultures. Annu Rev Public Health..

[3] Dewey C. A stunning map of depression rates around the world: The Washington Post; 2013. p. 7.

[4] Ezzati M, Vander Hoorn S, Lopez AD, Danaei G, Rodgers A, Mathers CD, Murray CJ (2006). Comparative quantification of mortality and burden of disease attributable to selected risk factors. Glob Burden Dis Risk Factors..

[5] Tomlinson M, Grimsrud AT, Stein DJ, Williams DR, Myer L (2009). The epidemiology of major depression in South Africa: results from the South African Stress and Health study: mental health. S Afr Med J..

[6] Hailemariam S, Tessema F, Asefa M, Tadesse H, Tenkolu G (2012). The prevalence of depression and associated factors in Ethiopia: findings from the National Health Survey. Int J Ment Heal Syst..

[7] Bedaso A, Kediro G, Yeneabat T (2018). Factors associated with depression among prisoners in southern Ethiopia: a cross-sectional study. BMC Res Notes..

[8] Cuijpers P, van Straten A, Warmerdam L, Andersson G (2009). Psychotherapy versus the combination of psychotherapy and pharmacotherapy in the treatment of depression: a meta-analysis. Depress Anxiety..

[9] Pagnin D, de Queiroz V, Pini S, Cassano GB (2004). Efficacy of ECT in depression: a meta-analytic review. J ECT..

[10] Frank E, Karp JF, Rush AJ (1996). Efficacy of treatments for major depression. Psychopharmacol Bull..

[11] Edition F (2013). Diagnostic and statistical manual of mental disorders. Am Psychiatric Assoc.

[12] Dadi AF, Miller ER, Bisetegn TA, Mwanri L (2020). Global burden of antenatal depression and its association with adverse birth outcomes: an umbrella review. BMC Public Health..

[13] Malhi GS, Mann JJ (2018). Course and prognosis. Lancet..

[14] Cuijpers P, Dekker J, Hollon SD, Andersson G (2009). Adding psychotherapy to pharmacotherapy in the treatment of depressive disorders in adults: a meta-analysis. J Clin Psychiatry..

[15] National Institute of Mental Health (US). Depression, what Every Woman Should Know: National Institutes of Health, National Institute of Mental Health; 1995.

[16] Annelies W, Sanne R, Daan C, Ad V, Arnt FAS, Gerben JW (2022). Deconstructing recovery: A prospective study on well-being, symptom severity and acceptance in patients with major depressive disorders. J Affect Dis..

[17] Davidson L (2005). Recovery, self-management and the expert patient: Changing the culture of mental health from a UK Perspective. J Ment Health..

[18] Bonney S, Stickley T (2008). Recovery and mental health: A review of the British literature. J Psychiatr Ment Health Nurs..

[19] Ramon S, Healy B, Renouf N (2007). Recovery from mental illness as an emergent concept and practice in Australia and the UK. Int J Soc Psychiatry..

[20] Masand PS (2003). Tolerability and adherence issues in antidepressant therapy. Clin Ther..

[21] Ashton AK, Jamerson BD, Weinstein WL, Wagoner C (2005). Antidepressantrelated adverse effects impacting treatment compliance: results of a patient survey. Curr Ther Res Clin Exp..

[22] Burra TA, Chen E, McIntyre RS, Grace SL, Blackmore ER, Stewart DE (2007). Predictors of self-reported antidepressant adherence. Behav Med..

[23] Fortney JC, Pyne JM, Edlund MJ, Stecker T, Mittal D, Robinson DE, Henderson KL (2011). Reasons for antidepressant nonadherence among veterans treated in primary care clinics. J Clin Psychiatry..

[24] Coleman R (1999). Recovery: An alien concept.

[25] Sayce L (2000). From psychiatric patient to citizen.

[26] Curry J, Silva S, Rohde P (2011). Recovery and recurrence following treatment for adolescent major depression. Arch Gen Psychiatry..

[27] Novick D, Montgomery W, Vorstenbosch E, Moneta MV, Dueñas H, Haro JM (2017). Recovery in patients with major depressive disorder (MDD): results of a 6-month, multinational, observational study. Patient Prefer Adherence.

[28] World Health Organization. WHO report on the global tobacco epidemic, 2008: the MPOWER package: World Health Organization; 2008. p. 11.

[29] Klein JP, Moeschberger ML (2003). Survival analysis: techniques for censored and truncated data.

[30] Cox DR (1972). Regression models and life-tables. J Roy Stat Soc: Ser B (Methodol)..

[31] Vaupel JW, Manton KG, Stallard E (1979). The impact of heterogeneity in individual frailty on the dynamics of mortality. Demography..

[32] Grant BF, Goldstein RB, Smith SM (2015). The Alcohol Use Disorder and Associated Disabilities Interview Schedule-5 (AUDADIS-5): reliability of substance use and psychiatric disorder modules in a general population sample. Drug Alcohol Depend..

[33] Blanco C, Compton WM, Saha TD (2017). Epidemiology of DSM-5 bipolar I disorder: results from the National Epidemiologic Survey on Alcohol and Related Conditions-III. J Psychiatr Res..

[34] Hasin DS, Shmulewitz D, Stohl M (2015). Procedural validity of the AUDADIS-5 depression, anxiety and post-traumatic stress disorder modules. Drug Alcohol Depend..

[35] Hasin DS, Greenstein E, Aivadyan C (2015). The Alcohol Use Disorder and Associated Disabilities Interview Schedule-5 (AUDADIS-5): procedural validity of substance use disorders modules through clinical re-appraisal in a general population sample. Drug Alcohol Depend..

[36] Goethals K, Janssen P, Duchateau L (2008). Frailty models and copulas: similarities and differences. J Appl Stat..

[37] Rey J, editor. IACAPAP Textbook of Child and Adolescent Mental Health: 2015 Edition: International Association for Child and Adolescent Psychiatry and Allied Professions; 2015.

[38] Hosmer DW, Lemeshow S. Applied survival analysis: time-to-event: Wiley Interscience; 1999. p. 21.

[39] Kaplan EL, Meier P (1958). Nonparametric estimation from incomplete observations. J Am Stat Assoc..

[40] Breslow NE (1975). Analysis of survival data under the proportional hazards model. Int Stat Rev/Revue Internationale de Statistique..

[41] Therneau TM, Grambsch PM (2000). The cox model. Modeling survival data: extending the Cox model.

[42] Duchateau L, Janssen P (2004). Penalized partial likelihood for frailties and smoothing splines in time to first insemination models for dairy cows. Biometrics..

[43] Wienke A. Frailty models in survival analysis: Chapman and Hall/CRC; 2010. p. 26.

[44] Hougaard P (1984). Life table methods for heterogeneous populations: distributions describing the heterogeneity. Biometrika..

[45] Novick D, Montgomery W, Vorstenbosch E, Moneta MV, Dueñas H, Haro JM (2017). Recovery in patients with major depressive disorder (MDD): results of a 6-month, multinational, observational study. Patient Prefer Adherence..

[46] Centers for Disease Control and Prevention (CDC) (2010). Current depression among adults. United States, 2006 and 2008. MMWR Morb Mortal Wkly Rep.

[47] Hanlon C, Medhin G, Alem A, Araya M, Abdulahi A, Hughes M, Tesfaye M, Wondimagegn D, Patel V, Prince M (2008). Detecting perinatal common mental disorders in Ethiopia: validation of the self-reporting questionnaire and Edinburgh Postnatal Depression Scale. J Affect Disord..

[48] Bulloch AG, Williams JV, Lavorato DH, Patten SB (2009). The relationship between major depression and marital disruption is bidirectional. Depress Anxiety..

[49] Yeshaw Y, Mossie A (2017). Depression, anxiety, stress, and their associated factors among Jimma University staff, Jimma, Southwest Ethiopia, 2016: a cross-sectional study. Neuropsychiatr Dis Treat..

[50] Gu L, Xie J, Long J, Chen Q, Chen Q, Pan R, Yan Y, Wu G, Liang B, Tan J, Xie X (2013). Epidemiology of major depressive disorder in mainland china: a systematic review. PLoS One..

[51] Mogga S, Prince M, Alem A, Kebede D, Stewart R, Glozier N, Hotopf M (2006). Outcome of major depression in Ethiopia: population-based study. Br J Psychiatry..

[52] Deyessa N, Berhane Y, Alem A, Hogberg U, Kullgren G (2008). Depression among women in rural Ethiopia as related to socioeconomic factors: a community-based study on women in reproductive age groups. Scand J Public Health..

[53] Damena T, Mossie A, Tesfaye M (2011). Khat chewing and mental distress: a community based study, in jimma city, southwestern ethiopia. Ethiop J Health Sci..

[54] Mekonnen E, Esayas S. Correlates of mental distress in Jimma town, Ethiopia. Ethiop J Health Sci. 2003;13(1).

[55] Welu SG, Aregawi DH, Gebreslassie HT, Kidanu KG. Prevalence and associated factors of depressive disorder among prisoners in Mekelle General Prison Center, Tigray, Ethiopia: a cross-sectional study design. Depress Res Treat. 2021;2021. 10.1155/2021/1942674 PMC8187058 34158975

[56] Goldman LS, Nielsen NH, Champion HC, Council on Scientific Affairs, American Medical Association (1999). Awareness, diagnosis, and treatment of depression. J Gen Int Med..

[57] Egede LE (2007). Major depression in individuals with chronic medical disorders: prevalence, correlates and association with health resource utilization, lost productivity and functional disability. Gen Hosp Psychiatry..

[58] Kader Maideen SF, MohdSidik S, Rampal L, Mukhtar F (2014). Prevalence, associated factors and predictors of depression among adults in the community of Selangor, Malaysia. PloS one..

